# Factors affecting utilization of modern contraceptive methods among women of reproductive age in Ethiopia

**DOI:** 10.1371/journal.pone.0294444

**Published:** 2023-11-16

**Authors:** Kassu Mehari Beyene, Sara Abera Bekele, Meseret Kassahun Abu

**Affiliations:** Department of Statistics, College of Natural Sciences, Wollo University, Dessie, Ethiopia; UiA: Universitetet i Agder, AUSTRALIA

## Abstract

**Introduction:**

Modern contraceptive use is important for improving health and socioeconomic outcomes, but Ethiopia is among the lowest-using countries. Therefore, this study aimed to determine factors affecting modern contraceptive use among women of reproductive age in Ethiopia.

**Methods:**

This population-based cross-sectional study used data obtained from the 2019 Ethiopia Mini Demographic and Health Survey (EMDHS). A total of 8,885 reproductive-age women were included in the analysis. A weighted generalized estimating equation approach was used to account for the clustering and weighting effects in the assessment of associations between modern contraceptive usage and socioeconomic and demographic variables.

**Results:**

Modern contraceptive use among women of reproductive age in Ethiopia is low (28%). Prevalence is highest among women aged 25-34 (40.11%), with higher education (30.97%), who are Orthodox Christians (31.67%), married (40.40%), middle wealth index (31.70%), female-headed households (31.42%), with 1-3 living children (44.85%), who headed by under 31 years old (40.07%), and in the Amhara region (34.45%). In the generalized estimating equation analysis, women aged 35-44 and over 45, Muslims, households heads aged 41-50 and over 50, and in female-headed households were less likely to use modern contraceptives, while women with primary, secondary, and higher education, married, middle and rich wealth index, and with 1-3 and more living children were more likely to use modern contraceptive than their counterparts (reference group) and were statistically significant.

**Conclusion:**

Modern contraceptive use is notably low among women of reproductive age in Ethiopia. Factors such as age, women’s educational level, religion, marital status, number of living children, wealth status, gender and age of household head, and region were identified as significant factors associated with modern contraceptive use. Therefore, to increase modern contraceptive use, governmental and non-governmental organizations should invest in women’s education and financial empowerment and raise awareness about the benefits of modern contraceptives, especially among older, unmarried, financially poor, elderly-led households, with few living children, and uneducated women.

## Introduction

Family planning is the practice of regulating the number, timing, and spacing of pregnancies through the use of modern contraceptives or other methods of birth control. Family planning is essential for empowering couples and individuals to make their own choices about their reproductive health and well-being. It not only reduces unintended pregnancies, high-risk pregnancies, and maternal and infant mortality, but also improves the nutrition and health of children, and leads to improved schooling and economic outcomes, particularly for women and girls [1]. Contraceptive use increased in all regions between 1990 and 2019. In 2019, 49% of women of reproductive age globally used some form of contraception, an increase of about 7% from 1990. Contraceptive use among women of reproductive age in sub-Saharan Africa increased from 13% in 1990 to 29% in 2019, one of the highest increases compared to other regions. During the same period, Ethiopia’s utilization rate increased from 3% to 26%. Among contraceptive users, the vast majority use modern methods. Globally, in 2019, 45% of women of reproductive age were using a modern method of contraception, which is equivalent to 91% of all contraceptive users. In 2019, modern contraceptive use in sub-Saharan Africa and Ethiopia was around 28% and 26%, respectively, accounting for 87% and 98% of all contraceptive users, respectively [1].

In 2019, there were 1.9 billion women aged 15-49 worldwide, with 1.1 billion having family planning needs. These women either use contraceptives (842 million modern and 80 million traditional methods) or have an unmet need (190 million want to avoid pregnancy but don not use contraceptives) [2]. In Africa, an estimated 139 million women of reproductive age want to avoid pregnancy, and 58 million of these women have an unmet need for modern contraceptive methods. Fulfilling this unmet need would reduce unwanted pregnancies by 78% (from 27 million to 6 million) and unsafe abortions by 78% (from 8.3 million to 1.8 million) and decrease maternal and neonatal mortality rates by nearly a quarter, even without improvements in maternal and neonatal care [3].

In Ethiopia, 44% of women of reproductive age, approximately 11.7 million, want to avoid pregnancy. Among them, 61% (7.2 million) use modern contraceptives, leaving 39% (4.5 million) with unmet needs. Among those with unmet needs, 4.4 million use no contraceptive methods, while 143,000 rely on less effective traditional methods. Meeting this unmet need could reduce the estimated 2.1 million unintended pregnancies by 90%, dropping unplanned births from 1.2 million to 128,000 and miscarriages from 571,000 to 59,000 annually. Providing modern contraceptives and proper care for pregnant women and newborns could reduce maternal deaths by 81% (from 12,000 to 2,000) and newborn deaths by 85% (from 92,000 to 13,000) per year in Ethiopia [4].

Several previous studies have examined factors that affect the use of modern contraceptive methods. Generally, these factors can be categorized into socioeconomic, demographic, and cultural factors. According to studies, factors such as women’s education level [5–7], women’s age [6–8], marital status [6], number of living children [5, 9], religion [7], place of residence [7, 8], wealth index [5, 9, 10], exposure to family planning messages [7, 9, 11], occupational status [6, 10, 12], highest educational level of husband [6, 7], attitude towards contraceptive [13], and knowledge of any contraceptive method [8] are associated with modern contraceptive use. In addition, some studies have shown that maternal health care services utilization, such as antenatal visits [12], postnatal care utilization [6], and health facility delivery [6] are associated with the use of modern contraceptives.

In order to increase modern contraceptive use in Ethiopia, the government is committed to ensure accessibility. Nevertheless, usage is still among the lowest in the world. As a result, several national and regional studies have been conducted to identify factors that affect contraceptive use [5, 8–10, 13–15], which is a critical component of effective family planning solutions. Most studies, particularly those conducted nationwide, used secondary data from the Ethiopian Demographic and Health Surveys [5, 8–10, 14]. Although each subject was given a specific sample weight and subjects were clustered in these surveys, to the best of our knowledge, none of the literature that has already been published has taken these factors into consideration when conducting analyses. The current study attempts to fill these gaps by modeling contraceptive utilization using the generalized estimating equation approach, which takes the correlated nature of the data into account, as this method is appropriate for obtaining an accurate inference and efficient estimate for the parameters of interest. The main aim of this study was to determine factors that affect the use of modern contraceptive methods among women of reproductive age in Ethiopia.

## Methods

### Study design and data source

In this population-based cross-sectional study, we used secondary data obtained from the 2019 Ethiopia Mini Demographic and Health Survey (EMDHS), which was made available by the Central Statistical Agency of Ethiopia. This is the second EMDHS and the fifth Demographic and Health Survey implemented in Ethiopia. The main objective of the survey was to provide up-to-date estimates for the health and demographic variables of interest at the national and regional levels and for urban and rural areas. The survey was conducted from March 21, 2019, to June 28, 2019, based on a nationally representative sample. To generate a nationally representative sample of households, two-stage stratified sampling was used. The first stage involved selecting clusters (i.e. enumeration areas) from recent nationwide survey sampling frames using probability proportional to cluster size, and the second stage involved selecting households in each cluster using an equal probability systematic selection method. For more details about the sampling method, the reader is referred to [16]. In the 2019 EMDHS, a total of 9,150 households were selected for the sample, from which 9,012 eligible women were interviewed in the survey. Due to incomplete information about some subjects, our study utilized the data from 8,885 women of reproductive age for which complete information was collected.

### Study variables and definitions

#### Outcome variable

The outcome variable was the use of modern contraceptive methods by Ethiopian women of reproductive age, which was classified as “Yes / No”. It was based on the question of what type of contraceptive method is currently using to delay or avoid pregnancy. There were four possible answers to this question: “no method,” “folkloric method,” “traditional method,” and “modern method”. This resulted in the development of a binary outcome variable, with women who are currently using modern contraceptives classified as “Yes” and those who are not classified as “No”. For more details about the types and definitions of contraceptive methods, the reader is referred to [16].

#### Predictor variables

**Fig 1:**
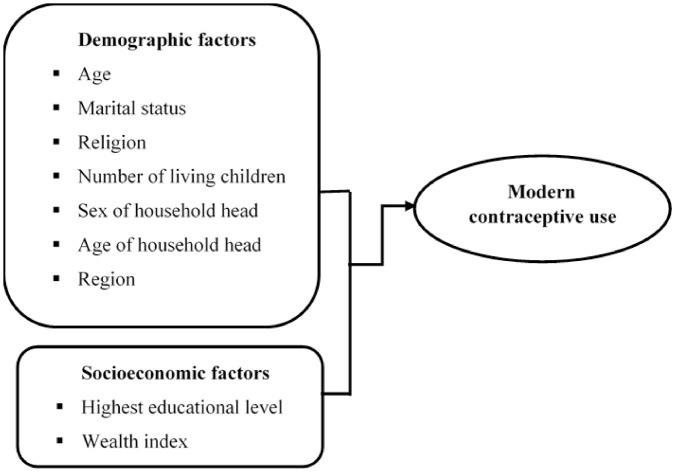
Conceptual framework to determine factors affecting modern contraceptive use.

The predictor variables considered in this study fall into two categories: socioeconomic and demographic. The literature on the determinants of contraceptive method utilization guided the selection of predictors. Age, highest educational level, religion, marital status, number of living children, wealth index, sex of household head, age of household head, and region were the nine predictor variables considered. Fig 1 shows a conceptual framework that can be used to study the relationship between modern contraceptive use and socioeconomic and demographic factors.

### Statistical analysis

In this study, the statistical analysis were conducted using the statistical software STATA version 16.0 and SAS version 9.4. In this study we employed both descriptive and inferential statistical methods. In order to summarize the distribution of socioeconomic and demographic characteristics of the respondents and utilization of modern contraceptive, frequencies and proportions were used. It is important to consider the features of the data, as well as the way the data was collected, when performing any statistical analysis. Clustered data is a type of data that occurs when the data collection methods or experimental design group the observations together. In this data, observations within the same cluster are more similar to each other than observations from different clusters. This forms correlation between observations within a cluster. In order to obtain an accurate inference and efficient estimate for the parameters of interest, it is necessary to account for this correlation using appropriate analysis methods. The generalized estimating equation approach introduced by [17] enables analysis of data collected in longitudinal or clustered designs. The generalized estimating equation models the mean response and the within-cluster association separately, assuming that the former is of primary interest while the latter needs to be considered in order to make valid inferences and the estimates more efficient. This approach involves specification of the form of correlation of responses within subjects or nested within cluster in the sample, that allows the generalized estimating equation to estimate models that account for the correlation of the responses [17]. It is suggested that, in the absence of logical ordering for observations within a cluster, an exchangeable correlation matrix should be used [18]. This correlation structure is better suited to situations where data are clustered within a particular subject but are not time-series data [19]. Therefore, this study used the generalized estimating equation model with exchangeable working correlation matrix to identify the determinants of the use of modern contraceptive methods in women of reproductive age. Another critical issue in statistical analysis of survey data is failing to account for the weights assigned to each sample unit, which can significantly bias the results of the analysis, leading to erroneous conclusions [20]. As a result, in this study, all of the reported figures were computed taking the sample weight into account. Variables with a p-value less than or equal to 0.05 were considered as significantly associated with utilization of modern contraceptive methods.

### Ethical consideration

This study used the 2019 Ethiopia Mini Demographic and Health Survey data which is available to the general public by request in different formats from the Measure DHS website (https://www.dhsprogram.com/data/). The survey data do not contain all identifying information. The authors applied to the Measure DHS by briefly stating the objectives of the study and got the permission to download data set. This study does not require ethical approval since the data is secondary and publicly available.

## Results

### Socioeconomic and demographic characteristics of respondents and modern contraceptive use

The socioeconomic and demographic characteristics distribution of women who use a modern contraceptive method is presented in Table 1. A total of 8,885 women in reproductive age were included in the analysis, and 28%(2495) of them use modern contraceptive methods. Of the total respondents, 41.5%(3691) were between 15 and 24 years old, 41.7%(3701) had primary education, 41.5%(3685) were Orthodox Christians, 64.6%(5743) were married, 34.7%(3083) had no living children, 46.8%(4161) were in the highest wealth quintile, 79.4%(7050) were from households with a male head of household, 28.7%(2550) were from households with a head of household aged 31-40, and 37.7%(3347) were from the Oromia region.

**Table 1:** Distribution of socioeconomic and demographic characteristics of women in reproductive age by their modern contraceptive use status.

Variables	Categories	Modern contraceptive use		Total
		No Frequency(%)	Yes Frequency(%)	Frequency(%)
**Age**	<25	2969(80.44)	722(19.56)	3691(41.54)
	25-34	1693(59.89)	1134(40.11)	2827(31.82)
	35-44	1242(68.85)	562(31.15)	1804(20.31)
	>44	486(86.32)	77(13.68)	563(6.34)
**Highest educational level**	No education	2597(72.36)	992(27.64)	3589(40.40)
	Primary	2642(71.39)	1059(28.61)	3701(41.65)
	Secondary	802(73.71)	286(26.29)	1088(12.25)
	Higher	349(69.03)	157(30.97)	507(5.70)
**Religion**	Orthodox	2518(68.33)	1167(31.67)	3685(41.47)
	Protestant	1674(68.75)	761(31.25)	2435(27.41)
	Muslim	2094(79.92)	526(20.08)	2620(29.48)
	Others	104(71.72)	41(28.28)	145(1.64)
**Marital status**	Never married	2278(97.98)	47(2.02)	2325(26.17)
	Merried	3423(59.60)	2320(40.40)	5743(64.64)
	Others	689(84.33)	128(15.67)	817(9.19)
**Number of living children**	0	2841(92.15)	242(7.85)	3083(34.70)
	1–3	1645(55.15)	1338(44.85)	2983(33.57)
	>3	1904(67.54)	915(32.46)	2819(31.73)
**Wealth index**	Poor	2347(76.90)	705(23.10)	3052(34.35)
	Middel	1142(68.30)	530(31.70)	1672(18.81)
	Rich	2901(69.72)	1260(30.28)	4161(46.84)
**Sex of household head**	Male	4835(68.58)	2215(31.42)	7050(79.35)
	Female	1555(84.74)	280(15.26)	1835(20.65)
**Age of household head**	<=30	1249(59.93)	835(40.07)	2084(23.45)
	31-40	1659(65.06)	891(34.94)	2550(28.70)
	41–50	1649(77.56)	477(22.44)	2126(23.93)
	>50	1833(86.26)	292(13.74)	2125(23.92)
**Region**	Somali	408(97.14)	12(2.86)	420(4.73)
	Tigray	464(73.77)	165(26.23)	629(7.08)
	Afar	76(89.41)	9(10.59)	85(0.96)
	Amhara	1328(65.55)	698(34.45)	2026(22.80)
	Oromia	2428(72.54)	919(27.46)	3347(37.67)
	Benishangul	73(74.49)	25(25.51)	98(1.11)
	SNNPR	1174(68.86)	531(31.14)	1705(19.19)
	Gambela	31(75.61)	10(24.39)	41(0.46)
	Harari	22(81.48)	5(18.52)	27(0.30)
	Addis Abab	333(75.34)	109(24.66)	442(4.97)
	Dire Dawa	53(81.54)	12(18.46)	65(0.73)
**Overall**		**6390(71.92)**	**2495(28.08)**	**8885(100)**

The prevalence of modern contraceptive use among women in the age group 15–24, 25–34, 35–44, and over 45 year old were 19.56%(322), 40.11%(1134), 31.15%(562), and 13.68%(77), respectively. The utilization among women with no education, primary education, secondary education and higher education were 27.64%(922), 28.61%(1059), 26.29%(286), and 30.97%(157), respectively. Among Orthodox, Protestants, Muslims, and followers of other religions, the prevalence of contraceptives use were 31.67%(1167), 31.25%(761), 20.08%(526), and 28.28%(41), respectively. A higher prevalence of modern contraceptive use was found among married women, 40.40%(2320) compared to unmarried, 2.02%(47), and “Others” (i.e. separated/divorced/widowed), 15.67%(128). Among women who have 1-3 living children, 44.85%(1338) use modern contraceptive method which is higher than women who have no children, 7.85%(242), and those with more than three living children, 32.46%(915). In terms of wealth status, the prevalence was about 23% (705) among poor, 32% (530) among middle, and 30% (1260) among rich women. The utilization for women in female- and male-headed households were about 31%(2215) and 15%(280), respectively. The prevalence of modern contraceptive use among women whose household head is under 31 years old, 40.07%(835), is higher than women in households where the head is 31-40, 34.94%(891), 41-50, 22.44%(477), and over 50, 13.74%(292), years old. In terms of prevalence by region: 34.45%(698) in Amhara, 31.14%(531) in SNNPR (Southern Nations, Nationalities and Peoples Region), 27.46%(919) in Oromia, 26.23%(165) in Tigray, 25.51%(25) in Benishangul-Gumuz, 24.66%(109) in Addis Ababa, 24.39%(10) in Gambela, 18.52%(5) in Harari, 18.46%(12) in Dire Dawa, 10.59%(9) in Afar, and 2.86%(12) in Somali.

### Determinants of modern contraceptive use among women of reproductive age

The predictor variables; age, highest educational level, religion, marital status, number of living children, wealth index, sex of household head, age of household head and region were considered in fitting a generalized estimating equation with exchangeable working correlation structure. The results of the final fitted generalized estimating equation analysis presented in Table 2 shows that age, highest educational level, religion, marital status, number of living children, wealth index, sex of household head, age of household head and region were all significantly associated with utilization of modern contraceptive methods. For the sake of brevity, we would like to emphasize in the following interpretations that all conclusions about the effect of a particular predictor variable on a response are based on the assumption that the remaining predictor variables in the model have been kept constant.

**Table 2:** Predictors of current utilization of modern contraceptive methods among women of reproductive age group in Ethiopia.

Variables	Categories	Estimated Odds	Std.Err.	z	p-value	95% CI for odds ratio	
		ratio				Lower	Upper
**Age**	<25	1.000					
	25-34	0.799	0.105	-1.710	0.087	0.619	1.033
	35-44	0.529	0.112	-3.010	0.003	0.350	0.801
	>=45	0.210	0.058	-5.650	<0.001	0.123	0.361
**Highest educational level**	No education	1.000					
	Primary	1.509	0.166	3.730	<0.001	1.216	1.872
	Secondary	1.971	0.358	3.740	<0.001	1.382	2.813
	Higher	2.168	0.639	2.620	0.009	1.216	3.863
**Religion**	Orthodox	1.000					
	Protestant	0.793	0.137	-1.340	0.179	0.565	1.112
	Muslim	0.527	0.113	-3.000	0.003	0.347	0.802
	Others	0.823	0.279	-0.570	0.566	0.424	1.600
**Marital status**	Never married	1.000					
	Married	18.580	4.402	12.330	<0.001	11.678	29.562
	Others	6.311	1.887	6.160	<0.001	3.512	11.340
**Number of living children**	0	1.000					
	1-3	3.414	0.574	7.300	<0.001	2.455	4.747
	>=4	4.257	0.846	7.290	<0.001	2.884	6.284
**Wealth index**	Poor	1.000					
	Middle	1.322	0.138	2.670	0.008	1.077	1.622
	Rich	1.286	0.148	2.190	0.029	1.027	1.610
**Sex of household head**	Male	1.000					
	Female	0.714	0.089	-2.710	0.007	0.560	0.911
**Age of household head**	<=30	1.000					
	31-40	0.793	0.098	-1.880	0.060	0.623	1.010
	41-50	0.701	0.121	-2.060	0.040	0.499	0.983
	>50	0.575	0.096	-3.330	0.001	0.415	0.796
**Region**	Somali	1.000					
	Tigray	4.688	2.968	2.440	0.015	1.356	16.212
	Afar	2.333	1.517	1.300	0.192	0.653	8.342
	Amhara	12.240	7.745	3.960	<0.001	3.541	42.307
	Oromia	9.410	5.608	3.760	<0.001	2.926	30.259
	Benishangul	8.745	5.206	3.640	<0.001	2.723	28.085
	SNNPR	10.661	6.480	3.890	<0.001	3.239	35.088
	Gambela	4.671	3.115	2.310	0.021	1.264	17.261
	Harari	5.946	3.554	2.980	0.003	1.843	19.189
	Addis Ababa	8.507	5.301	3.440	0.001	2.508	28.852
	Dire Dawa	5.809	3.466	2.950	0.003	1.804	18.704

The estimated odds ratios (OR) for the age groups 35-44, and above 45 were 0.529 [95% CI: 0.350-0.801] and 0.210 [95% CI: 0.123-0.361], respectively, when compared to those younger than 25. This means that the use of modern contraceptive methods by women aged 35-44, and over 45 is lower by about 47 percent, and 79 percent, respectively, than by women aged under 25. Women with primary, secondary and higher education were 1.509 [OR = 1.509, 95% CI: 1.216-1.872], 1.971 [OR = 1.971, 95% CI: 1.382-2.813] and 2.168 [OR = 2.168, 95% CI: 1.216-3.863], respectively, times more likely to use modern contraceptive methods compared to women with no education. In comparison to Orthodox religion follower women, Muslim religion follower women are about 47 percent [OR = 0.527, 95% CI: 0.347-0.802] less likely to use modern contraceptives methods. In contrast, there was no significant difference in the likelihood of women using modern contraceptives between Orthodox and Protestants and followers of other religions. Married women and “Others” were about 18 [OR = 18.580, 95% CI: 11.678-29.562] and 6 [OR = 6.311, 95% CI: 3.512-11.340] times more likely to use modern contraceptive methods compared to those women who never married. There was nearly three-fold more chance in contraceptive use among women who had one to three living children and about four-fold more chance among those who had four or more living children compared to women without children [OR = 3.414, 95% CI: 2.455-4.747] and [OR = 4.257, 95% CI: 2.884-6.284]. Women whose household wealth index is middle, and rich are 1.32 [OR = 1.322, 95% CI: 1.077-1.622], and 1.29 [OR = 1.286, 95% CI: 1.027-1.610] times more likely to use modern contraceptives than women whose wealth index is the least. In households headed by women, the likelihood of using contraceptive methods was about 29 percent lower [OR = 0.714, 95% CI: 0.560-0.911] than that for women in households headed by men. Women in households with heads of household aged 41-50, and over 50 use contraceptives approximately 30% [OR = 0.701, 95% CI: 0.499-0.983 and 43% [OR = 0.575, 95% CI: 0.415-0.796] less likely than women in households with head of household under 30 years of age. Women living in Amhara, SNNPR, Oromia, Benishangul, Addis Ababa, Harari, Dire Dawa, Tigray, and Gambela, respectively, were about 12.24 [OR = 12.240, 95% CI: 3.541-42.307], 10.66 [OR = 10.661, 95% CI: 3.239-35.088], 9.41 [OR = 9.410, 95% CI: 2.926-30.259], 8.75 [OR = 8.745, 95% CI: 2.723-28.085], 8.51 [OR = 8.507, 95% CI: 2.508-28.852], 5.95[OR = 5.946, 95% CI: 1.843-19.189], 5.81 [OR = 5.809, 95% CI: 1.804-18.704], 4.69 [OR = 4.688, 95% CI: 1.356-16.212], and 4.67 [OR = 4.671, 95% CI: 1.264-17.261] times more likely to use modern contraceptive methods than that of women living in the Somali region.

## Discussion

The main objective of this study was to identify socioeconomic and demographic variables that affect the use of modern contraceptive methods among women in reproductive age in Ethiopia based on data from the 2019 Ethiopia Mini Demographic and Health Survey. The finding of this study revealed that about 28 percent of the study participants were currently using modern contraceptive methods, which is low. This result is in line with earlier studies conducted in Hadiya, Ethiopia (23.9%), Burkina Faso (23.6%) and Liberia (23.87%), which also found low prevalence rates [15, 21, 22]. The prevalence of modern contraceptive use, however, varies by socioeconomic and demographic characteristics of women of reproductive age in Ethiopia. The prevalence is highest among women 23-34 years old (40.11%), with higher education (30.97%), with 1-3 living children (44.85%), who are Orthodox Christians (31.67%), who are married (40.40%), from female-headed households (31.42%), who headed by under 31 years old (40.07%), and in the Amhara region (34.45%).

Moreover, this study revealed a significant relationship between age, highest educational level, religion, marital status, number of living children, age of household head, sex of household head, wealth index and region with modern contraceptive use among women of reproductive age. Compared with women under 25 years old, women in the age groups 35-44 and over 45 were less likely to use modern contraceptive methods. In contrast, the likelihood of using modern contraceptive methods was not significantly different between women under 25 and women 25-34 years old. The rate of using modern contraceptive methods among women 35-44 and over 45 years old is 47% and 79% lower, respectively, compared to women under 25 years old. This finding is consistent with other studies conducted in Ethiopia [9, 13, 14], Zambia [23] and countries in sub-Saharan Africa [7]. This can be explained by the fact that younger women are more aware of the benefits of modern contraceptive methods for both maternal and child health. Another possible explanation is that as women age, their likelihood of changing their beliefs or attitudes about contraception may decrease.

Regarding the highest level of education of respondents, this study indicates that women with primary, secondary and higher education level are more likely to use modern contraceptive methods than women with no education. This is consistent with the findings of other similar studies conducted in Ethiopia [8, 13], Kenya [11, 24], Uganda [25, 26], Liberia [22], and countries in sub-Saharan Africa [7]. The reason might be that women with higher education have more access to health information and are more likely to be positive about modern contraceptives.

This study also showed that religion is a significant predictor of modern contraceptive use among women of reproductive age. Muslim religion follower women are 47% less likely to use modern contraceptive method than those women who follow Orthodox religion. On the other hand, the results showed no significant difference in the likelihood of using modern methods of contraceptives between Orthodox and Protestant women and followers of other religions. This is consistent with similar studies conducted in Ethiopia [5, 14]. In relation to marital status, this study found that married women and “Others” (i.e. widowed women, separated women, or divorced women) are about 18 and 6 times more likely to use modern contraceptive methods than women who have never been married. This finding is supported by findings from the studies conducted in Ethiopia [27] and Uganda [25]. The possible explanation is that married women may have the desired number of children, which may influence contraceptive use. In addition, married women and “other” groups are more likely to have sexual intercourse than those women who have never married, so they can use modern methods of contraceptives to avoid unwanted pregnancies.

The number of children alive is also significantly associated with the use of modern contraceptives. In comparison with women without children, those with one to three living children and more than four children were three times more likely and four times more likely to use modern contraceptives. This was consistent with other findings that reported increased use of contraceptives as the number of living children increased [5, 13, 22, 25]. This might be because women with one or more children may feel that they need to avoid or limit childbirth more than women without children. In other words, women with few children need to give birth to reach the desired number of families, which can affect the use of modern contraceptives.

Another predictor associated with utilization of modern contraceptives by women of reproductive age is the household wealth index. There was a significant association between households with higher wealth and high contraceptive use. Women from households with middle, and rich wealth status are about 1.32, and 1.29 times more likely to use modern contraceptives than women with the poor wealth index. This is consistent with the findings of studies conducted in Ethiopia [5, 9, 14], Kenya [11], Uganda [26], Zambia [23], Nigeria [28], countries in sub-Saharan Africa [7], and countries in East Africa [6]. The possible explanation is that women in richer households might have better access to education, mass media and health services, and perhaps are more aware about modern contraceptives methods than those from poor households.

Age and gender of the household head are also significant predictors of modern contraceptive method use among women of reproductive age. Women in male-headed households are more likely to use modern contraceptive methods than women in female-headed households. Women in households with heads of households between 41 and 50 years old and over 50 years old are about 0.7 and 0.58 times less likely to use modern contraceptive methods than women in households with heads of households under 30 years old.

The study also showed that there is statistically significant association between the region of residence and utilization of modern contraceptive methods among women of reproductive age. Women in Amhara, SNNPR, Oromia, Benishangul, Addis Ababa, Gambela, Tigray, Dire Dawa, and Harari were more likely to use modern contraceptive methods than women in the Somali region. In contrast, there was no significant difference in the likelihood of using modern contraceptive methods between women in the Afar and Somali regions. This insignificant difference in contraceptive use among women in the Afar and Somali regions may be explained by the fact that these regions share similar cultures, faiths, and other traditions. These findings support previous studies that found regional variation in the likelihood of using modern contraceptive methods among women of reproductive age [5, 8].

The finding of this study has important implications on polices and practices of modern contraceptive use. Policymakers should put in place policies and efforts to support women to pursue higher education and empower financially to increase modern contraceptive use which will help to reduce unwanted pregnancies and abortions, and improve maternal and child health. Both governmental and non-governmental organizations should design family planning programs to reach older women, never married, with no living children, women headed by female household heads, and older household heads, as these could increase overall modern contraceptive use among women of reproductive age in Ethiopia. In addition, religious leaders should teach their followers about the importance of modern contraceptive methods to control their family size. Moreover, attention should be given to women in regions with low contraceptive prevalence; such as Afar, and Somali.

The main strength of the current study is that it used a nationally representative sample data set with a large sample size to produce a result that better reflects the current modern contraceptive use and its associated determinants. A further strength of this study is that both the sample weight and the clustered nature of the EMDHS data were considered during the analysis. The generalized estimating equation approach was used to accomplish this. It is important to note, however, that secondary data, such as the EMDHS, may not include some important variables that may influence the use of modern contraceptives. A further note about EMDHS is that it is based on self-reported data, so it may be vulnerable to recall biases and social desirability biases as is the case with any self-reported data.

## Conclusion

In this study, we found that the modern contraceptive use in Ethiopia among women of reproductive age group is low (28%). Age, highest educational level, religion, marital status, number of living children, wealth index, sex of household head, age of household head, and region were identified factors that significantly associated with the utilization of modern contraceptives in Ethiopia. Furthermore, the results indicated that the use of modern contraceptives increases as education level, number of living children and wealth status increase. In contrast, contraceptives use decrease with increase in women’s age and age of household head. Therefore, in order to increase the use of modern contraceptives in Ethiopia, several important interventions are needed from both the federal and regional governments; efforts are needed to get more resources and made available for women’s education and financial empowerment. Non-governmental organizations and civil society can play a role in raising social awareness about the advantages of using modern contraceptive methods, especially among older women, women with no education, financially poor women, elderly household heads, never married women and women with fewer number of living children.
